# Validation of trophic and anthropic underwater noise as settlement trigger in blue mussels

**DOI:** 10.1038/srep33829

**Published:** 2016-09-20

**Authors:** Aurélie Jolivet, Rejean Tremblay, Fréderic Olivier, Cédric Gervaise, Rémi Sonier, Bertrand Genard, Laurent Chauvaud

**Affiliations:** 1 Institut Universitaire Européen de la Mer (UMR CNRS 6539), Université de Bretagne Occidentale, rue Dumont d’Urville, F-29280 Plouzané, France; 2 TBM environnement/Somme, 115 rue Claude Chappe, Technopole Brest Iroise, F-29280 Plouzané, France; 3 Institut des sciences de la mer, Université du Québec à Rimouski, 310 allée des Ursulines, Rimouski, Québec, G5L 3A1, Canada; 4 Muséum national d’Histoire naturelle, (UMR BOREA 7208, CNRS/MNHN/UPMC/IRD), 61 rue Buffon, CP53, 75231 Paris Cedex 05, France; 5 Chaire CHORUS, fondation Grenoble INP, GIPSA-LAB, Grenoble INP et CNRS, 11 rue des Mathématiques, Domaine Universitaire, BP 46 F-38402 Saint Martin d’Heres cedex, France.; 6 Department of Fisheries and Oceans Canada, Gulf Fisheries Centre, Science Branch, P.O. Box 5030, Moncton, Nouveau-Brunswick, E1C 9B6, Canada

## Abstract

Like the majority of benthic invertebrates, the blue mussel *Mytilus edulis* has a bentho-pelagic cycle with its larval settlement being a complex phenomenon involving numerous factors. Among these factors, underwater noise and pelagic trophic conditions have been weakly studied in previous researches. Under laboratory conditions, we tested the hypothesis that picoplankton assimilation by the pediveliger blue mussel larvae acts as a food cue that interacts with anthropic underwater sound to stimulate settlement. We used ^13^C-labeling microalgae to validate the assimilation of different picoplankton species in the tissues of pediveliger larvae. Our results clearly confirm our hypothesis with a significant synergic effect of these two factors. However, only the picoeukaryotes strains assimilated by larvae stimulated the settlement, whereas the non-ingested picocyanobacteria did not. Similar positive responses were observed with underwater sound characterized by low frequency vessel noises. The combination of both factors (trophic and vessel noise) drastically increased the mussel settlement by an order of 4 compared to the control (without picoplankton and noise). Settlement levels ranged from 16.5 to 67% in 67 h.

The blue mussel, *Mytilus edulis*, like the majority of temperate marine benthic mollusks, produces free swimming planktotrophic larval stages, which feed, grow, and disperse via water currents until the competent pediveliger stage is physiologically capable of settling and metamorphosing onto a substrate^1^. However, pediveliger larva can extend the duration of its planktonic stages to significantly increase their size at metamorphosis^2^,^3^,^4^,^5^,^6^,^7^. Martel *et al*.^4^ observed that delayed metamorphosis can induce 47.8% of additional larval shell growth representing a 322% difference in larval body mass at settlement. Among the wide range of settlement cues interacting in the larval settlement behavior, the ambient underwater sound is the most probable for guiding onshore orientation by pelagic larvae of numerous common coastal species such as fish^8^,^9^, crustacean^10^,^11^ and coral larvae^12^. Moreover, ambient underwater sound from reefs has been shown to initiate settlement behavior and decrease metamorphosis initiation time on benthic dwelling larval stages in several crab species^13^,^14^. A recent study focusing on mytilid *Perna canaliculus* showed that larval settlement was significantly faster when exposed to the underwater noise produced by a passenger and freight ferry^15^. Over the last 50 years, vessels contributed to a 32-fold increase in the low frequency noise present in some parts of the ocean^16^. Vessel noise is generated by the operating high energy noisy propellers, gears and diesel generators^17^. The underwater sound produced by vessels is biologically important for settlement stage larvae, particularly species involved in fouling and bioinvasion such as mussels, ascidians and barnacles^15^,^18^,^19^.

In their perspective section, Wilkens *et al*.^15^ suggested that “*the relative importance of underwater sound as a settlement cue or the potential for underwater sound to act in a synergistic manner with other settlement cues is unknown*”. Field studies of Toupoint *et al*.^6^,^20^ suggested that picoplankton abundance acts as a trophic settlement trigger for mussel larvae and that these pelagic cues are dominant over other cues as substrate features including biofilms^21^. Pelagic picophytoplankton includes prokaryotes and eukaryotes that are classically defined by the 0.2–2 μm size range. Prokaryotic species belong mostly to cyanobacteria of the genus *Prochlorococcus*^22^ and *Synechococcus*^23^. The latter is a small unicellular cyanobacterium, 1 μm in diameter, abundant in temperate and tropical oceans^22^ and contributing to a substantial proportion of marine primary production^24^,^25^. Previous studies showed that when nanoplankton biomass is limited, invertebrates’ larvae may exert considerable grazing pressure on *Synechococcus* spp.^25^,^26^.

In the present study, we propose to analyze the synergistic or antagonistic effects of two interacting factors on larval settlement of the blue mussel (*Mytilus edulis*): i) the anthropic underwater sound and ii) the contribution of picoplankton (*Nannochloropsis oculata* and *Synechococcus* sp.). Experiment using ^13^C-labeled picoplankton was performed in parallel to test whether the picoeukaryotic species were ingested and assimilated by pediveliger larvae. Fatty acid trophic markers methods were used to validate the microalgal assimilation as mussels are not able to biosynthesizing highly polyunsaturated fatty acids^27^. We tested the hypothesis that picoplankton cells, inducing a nutritional cue, are assimilated by blue mussel pediveliger larvae and together with specific underwater sound, can stimulate settlement.

## Results

### Underwater sound recordings

**Figure 1:**
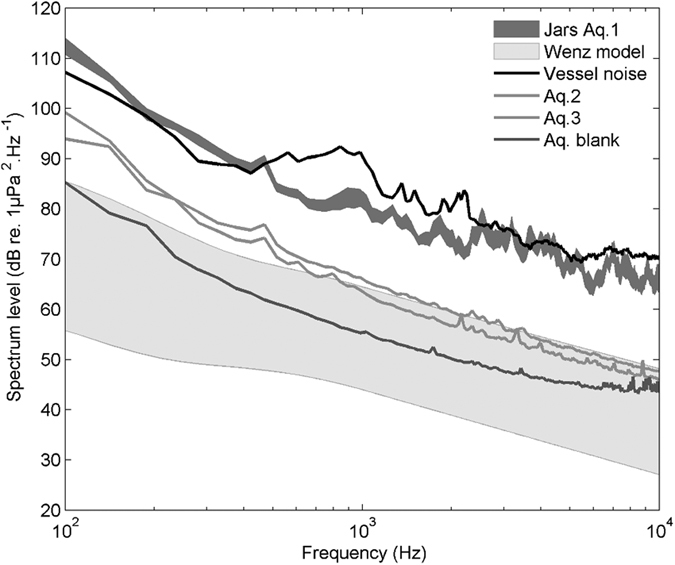
Spectrums (dB re 1 μPa^2^ Hz^−1^) of sounds when recorded: vessel noise recorded *in situ* (black line), in the sound treatment (dark gray area corresponding to the mean of the 10 jars ± 1 SEM), in the two adjacent aquariums with silent treatment (Aq. 2 and 3 in gray lines), and in the aquariums before experiment (Aq. Blank, dark gray line). The gray area corresponds to variation of natural ambient noise estimated from Wenz’ formula for different wind (wind speeds from 0 to 10 m s^−1^) and traffic conditions (traffic density from 1 to 7).

Replayed vessel noise in the different jars of Aquarium 1 was homogeneous and measured at 127 ± 3 dB re 1 μPa between 100 and 1,000 Hz corresponding to the *in situ* recorded source signal (Fig. 1). In the two others aquariums, corresponding to treatment without sound emission, the sounds levels differed from the Aquarium 1 (Fig. 1 and Table 1). The sound levels were slightly higher than the conditions before experiment (Table 1) but remained consistent with rough natural conditions defined by Wenz’ formula^28^ (Fig. 1).

### Settlement rate

**Figure 2:**
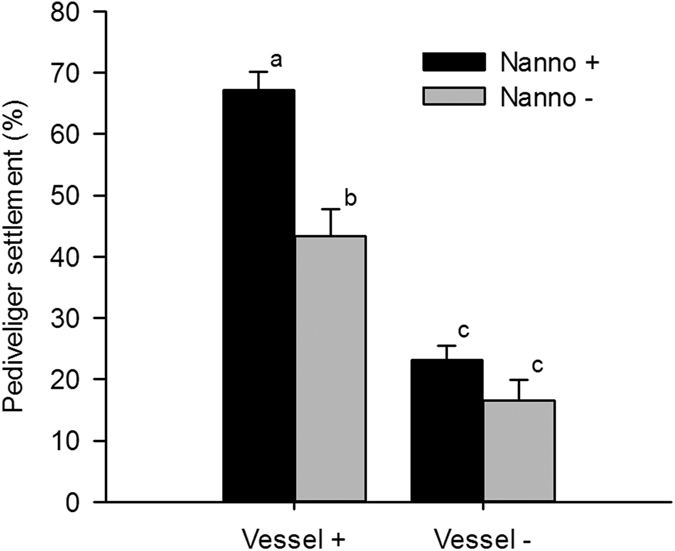
Estimated settlement percentage (mean ± SEM) for each condition: sound treatment (noted Vessel +/−) and trophic treatment with *Nannochloropsis oculata* (noted Nanno +/−). Letters indicate significant differences.

Vessel noise had an interactive significant with *N. oculata* trophic treatment (F_1, 16_ = 6.682, p = 0.02). Thus, adding *N. oculata* induced a significant increase of 6.6% of larval settlement rate in the silent aquarium, vessel sounds alone increased settlement by 27% in aquarium without *N. oculata*, and the most intense effect was observed with the combination of trophic and vessel noises (increase of 50.7% comparatively to control condition; Fig. 2). By contrast to *N. oculata*, the addition of *Synechococcus* sp., did not significantly affect the mussel settlement rate at a mean of 10.2 ± 1.8% similar to control (t_4_ = 0.556, p = 0.608).

### Larval Size and Metamorphosis

**Figure 3:**
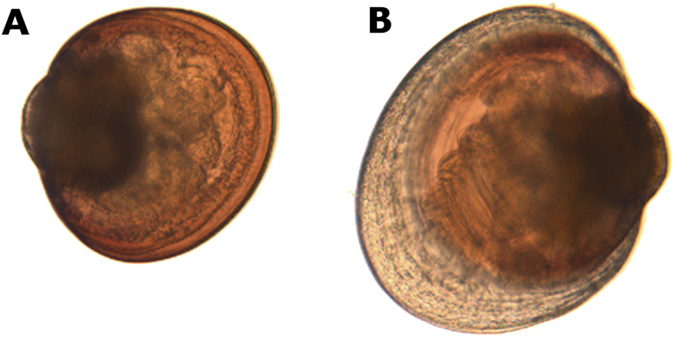
Photograph showing pediveliger larvae (A, 267 μm) and post-larvae with the prodissoconch II (B, 346 μm) of *Mytilus edulis* observed at a 100× magnification.

Neither the occurrence of vessels sounds nor *N. oculata* differentiate swimming larvae size (Table 2, vessel: F_1, 317_ = 1.05, p = 0.307; trophic: F_1, 317_ = 0.003, p = 0.955; interaction: F_1, 317_ = 0.13, p = 0.723). However there was a significant interaction between sound and trophic treatments against settlers size (F_1, 249_ = 4.45, p = 0.033) resulting in sizes 5% smaller in condition with vessel noises than other treatments (Table 2, p < 0.03). Metamorphosis was determined by the presence of a prodissoconch shell (Fig. 3). In swimming larvae, the mean metamorphosis success was very low or null for all treatments (0.9 ± 0.7%) comparatively to settlers larvae (45.7 ± 5.3%), suggesting that the larvae settled before engaging in the metamorphosis process. In settlers, vessels sounds and trophic treatments had no significant effect in the observed percentage of metamorphosis (Table 2, vessel: F_1, 19_ = 0.90, p = 0.357; trophic: F_1, 19_ = 1.17, p = 0.295; interaction: F_1, 19_ = 0.23, p = 0.639).

### Assimilation

Lipids of the two picoplankton species treatments (*N. oculata* and *Synechococcus* sp.) were strongly enriched in ^13^C by our culture method as the %^13^C of each FA analysed were over 87% (Table 3). Results of the Permanova analysis showed significant differences (Pseudo-F_(3–9)_ = 1698, p = 0.0002) between the four treatment (*N. oculata*, *Synechococcus* sp. and larvae fed with each picoplankton species) in the ^13^C enrichment for dominant FA (Table 3). The pair-wise tests indicate that all comparisons were significantly different (p < 0.001), except for *N. oculata* and *Synechococcus* sp. showing similar levels of ^13^C enrichment (t = 3.961, p = 0.055). Larvae fed with ^13^C *N. oculata* showed FA enrichment in their polar lipids largely over the level observed in larvae fed with ^13^C *Synechococcus* sp. with enrichment levels exceeding 21%, except for the 22:2 NMI with 13.5%. For larvae fed with ^13^C *Synechococcus* sp., the labeling levels were close to the natural levels of 1.1%.

## Discussion

Our results clearly confirm our hypothesis that picoplankton assimilation by blue mussel pediveliger larvae acts as food cues that interacts, with some degree of synergy, with anthropic underwater sound to stimulate larval settlement. Toupoint *et al*.^6^ demonstrated in natural conditions that *Mytilus edulis* larval settlement was enhanced in the presence of picoplankton, but could not determine whether the picoplankton acted as a chemical cue or was ingested and assimilated by the larvae. Our experimental study clearly confirms that only picoplankton species involved in the diet of pediveliger larvae act as a trophic settlement trigger (TST). Moreover, in accordance with a Wilkens *et al*.^15^ study on *Perna canaliculus*, we also demonstrated the positive response of vessel noise on *M. edulis* settlement. Although previously hypothesized by Wilkens *et al*.^15^, we show here for the first time that anthropic underwater noise act in a synergistic manner to amplify the TST’s impact: the combine synergic effects of these two factors greatly increased the settlement success up to 70% in less than 67 h. Such high settlement levels are rare in laboratories studies on the blue mussel. For instance, the settlement success of competent *M. edulis* larvae (>50% pediveliger) in a flow-through system with the best suitable hydrodynamic treatment after 7 days, was less than 20%^29^. In laboratory static conditions without filamentous substrate (as in the present study), no settlement success of *M. edulis* larvae^30^. Larval settlement of *M. edulis*, and bivalves in general, is complex and involves a wide range of interacting settlement cues like surface wettability and microtopography^31^,^32^, presence of conspecifics^33^, light regimes^33^,^34^, chemical cues^32^,^35^,^36^, biofilms^20^,^37^ and hydrodynamic conditions^29^,^38^. When tested in various combinations, some of these factors have shown greater success than others in supporting larval settlement, such as pelagic trophic condition compared to biofilm development^20^, while other combinations, such as filamentous substratum and turbulence^30^ have shown to be synergic. Our results show that the combination of anthropic vessel sound and trophic condition increased settlement success up to 51% than the control. In the present study, the anthropic and natural underwater sounds were recorded from one of the important suspension mussel culture areas of Eastern Canada, where about 38% of the St Peter’s Bay total surface area is dedicated to mussel farming. The natural underwater ambient sound was measured at levels inferior to the two aquariums corresponding to the conditions “silent” in our experiment. Ambient soundscape cultured suspension mussels differ clearly from surf-zones of rocky shores and biogenic reefs, which is the natural habitat of *M. edulis*^39^,^40^. Such habitats produce natural underwater sounds ranging from 88 to 145 dB re 1 μPa RMS including the sound level of vessel noise (130 dB re 1 μPa). Although the ambient underwater sound is now recognized as an important factor in guidance for the larvae, the possible sensory mechanism for the mussel larvae to detect sound is still unknown.

Anthropic underwater noise increases the settlement of mussel pediveliger larvae but decreases the size of the settler with potential cascading ecological impacts. Indeed, as the body mass follows the usual cubic progression in relation to shell length, a 5% smaller size could represent a much greater decrease of larval body mass. With marine benthic invertebrates, the size of the first post-larval stage is recognized as a key life history parameter, which constitutes a significant biological attribute in the ecology, ethology, and evolutionary biology of the species^41^,^42^,^43^,^44^,^45^. High post-larval mortality could reach 90% and is mostly related to predation, stress tolerance sensitivity, hydrodynamics condition and competition^46^,^47^,^48^.

Considering the results of isotopic labeling experiments, we showed that the fatty acids (FA) of *Synechococcus* spp. were weakly assimilated by the larvae, contrasting with the species *N. oculata*, as demonstrated by the very low level of enriched FA in the lipid polar fraction. Polar lipids are composed mainly of different phospholipid classes that are part of the cell membrane of bivalve larvae^49^. Nonetheless, our results clearly show that *N. oculata* was ingested and assimilated by pediveliger larvae: the 20 % increase of ^13^C enrichment in the polar FA of larvae fed with labeled *N. oculata* indicates that such microalga provided carbon in the cell membrane of pediveliger larvae during 72 h in experimental conditions. Moreover, the presence of ^13^C enriched 22:2 non methylene-interrupted (NMI) in the larvae, absent from the microalgae, suggest a biosynthesis of new FA in complement to the direct transfers from specific FA from *N. oculata* to polar lipids This NMI FA biosynthesis by mollusk have already been demonstrated in several bivalve species, including *M. edulis*^50^,^51^,^52^,^53^. These NMI are biosynthesized by the use of 16:1ω-7 and 18:1ω-9 as precursors then elongated to 20:1ω-7 and 20:1ω-9, desaturated into 20:2 and finally elongated in 22:2^53^. As mussels are able to synthesize *de novo* this NMI FA, such result gives additional evidence of both ingestion and assimilation of *N. oculata* by *M. edulis* larvae. With contrast to the *N. oculata* results, the fact that *Synechococcus* spp. did not contribute to the larval nutrition could be related to its smaller size (<1 μm) compared to *N. oculata* (around 2 μm). Hence, we suggest that the role of the trophic settlement trigger should be associated only to picoplankton species, most abundant in marine ecosystems^54^,^55^,^56^, that contribute to the nutrition of mussels’ larvae.

Picoplankton can reach very high concentrations and dominate the phytoplankton biomass in relatively nutrient rich coastal systems^54^,^55^,^56^. Moreover, since the last decade, the contribution of small-size cells to phytoplankton community has increased^57^,^58^. In the context of future predicted climate conditions, the biomass of dominant picoplankton species will more contribute to the primary production and their relative weight will change^59^. In parallel, future marine shipping and associated underwater noise will intensify, especially in low impacted systems as in the Arctic Ocean^60^. Synergetic effects between noise and picoplankton combined with global predicted increased levels should therefore deeply affect the mussel’s recruitment patterns in coastal areas.

## Methods

### Underwater sound

Recordings of underwater sound were made at St Peter’s Bay on Prince Edward Island (Canada, 46°25.963 N; 62°39.914 W), a major site of suspension mussel aquaculture, using 100-m longlines, with a calibrated hydrophone (High Tech, Inc., Mississippi, USA, HTI-99-HF: sensitivity −169.7 dB re 1 V/μPa; frequency range 2 Hz to 125 kHz flat response) installed at the base of the mussel line near the anchor (25 cm from the bottom) in the middle of the farm. The output was captured with a calibrated underwater acoustic recorder (RTSYS-Marine Technologies, France, EA-SDA14, 156 kHz, 24-bit resolution). The recording lasted 24 hours and was collected on July 5 to 6, 2013. To obtain a sound sequence with anthropic noise, the farmer’s boat (D & H Boatbuilding hull of 11 meters long) equipped with a diesel motor (Cummins 300 hp C series) was passed three times at a slow speed just above the experimental longline. All recordings were conducted in near calm conditions (0.2 m wave height and 3.8 m s^−1^ wind speed, http://climat.meteo.gc.ca/). Digital recordings were transferred to a PC to enable spectral composition and source sound level determination using the MATLAB (The MathWorks, Inc.) software with customized written codes for post-recording sounds analysis. From the recordings, a sequence lasting 30 s was selected corresponding to “vessel noise” at maximum sound intensity. Moreover, a sequence of 30 s per hour was analyzed to evaluate the ambient underwater noise without anthropic noise.

### Larval culture

Two years-old mature mussels collected from St Peter’s Bay were conditioned to spawn in the laboratory^61^ and the larvae were reared^62^. Introduction and transfer permits from Fisheries and Oceans Canada were obtained and our study did not involve endangered or protected species. Briefly, fertilized eggs (66.4 ± 7.7 μm) from 20 males and 20 females were reared at 20 °C and 28 PSU salinity in a final experimental concentration of 20 larvae ml^−1^ in 150 l of filtered seawater (1 μm pore-size). Two upwelling flow-through systems of 0.1 l min^−1^ with natural light period (16:8 h) were used. Larvae were fed continuously at 30 cell μl^−1^ with a 1:1:1 mixture of *Pavlova lutheri* (CCMP 1325), *T-isochrysis lutea* (CCMP 463) and *Chaetoceros muelleri* (CCMP 1316), named after PTC mixture. Microalgae were obtained from the National Center for Marine Algae and Microbiota, NCMA (Bigelow Laboratory for Ocean Sciences, East Boothbay, ME, USA) and cultured in seawater enriched with f/2 medium at 20 °C^63^. Survival rate was 52.2 ± 2.5% until 90% of population was at the pediveliger stage, and the daily growth was 10.2 ± 0.3 μm d^−1^.

### Larval settlement experiment

For the experiment, 40-l aquariums were used, each one containing 30 l of water and ten 250-ml individual jars placed on a platform at 18.5 cm from the aquarium bottom to keep jars rims 1 cm above the surface. Aquariums were used to maintain constant water temperature at 20 °C (±1 °C) monitored by probes (Onset Hobo Water Temp Pro V2 Data logger U22-001), with a natural light period (16:8 h). Each aquarium corresponded to an acoustic treatment and jars to a trophic treatment with 5 replicates per condition randomly placed on the platform. At the start of the experiment, 1 pediveliger larvae ml^−1^ were introduced in each jar filled with 240 ml of 1 μm filtered and UV treated seawater.

The experiment consisted of two aquariums for sound treatments, *i.e.* presence and absence of ‘vessel noise’”, where presence or absence of *Nannochloropsis oculata* (CCMP 525) was included. A third similar aquarium, without sound treatment, has been added to evaluate the presence/absence effect of a second species of picoplankton *Synechococcus* sp. (CCMP 1333). The jars corresponding to control for trophic treatments contained PTC mixture (1:1:1) at a total concentration of 36 cells μl^−1^. Concentrations in jars with *N. oculata* and *Synechococcus* sp. were adjusted to maintain the similar algae biomass than control estimated by dry mass on GF/F filter rinsed with ammonium formate (6%). Consequently, concentrations were 75 cells μl^−1^ for *N. oculata* or 150 cells μl^−1^ for *Synechococcus* sp. Larvae were left undisturbed for 67 hours. Each jar was lightly rinsed twice over a 200 μm sieve to collect unfixed larvae and the remainder of the jar was gently brushed to collect settled larvae and preserved in 10% formaldehyde. Swimming and settled larvae were counted under binocular microscope using Dollfus counting cell and size was determined using ImageJ, free image processing software, obtained with an upright Olympus BX-41 associated to high resolution video camera (Evolution VF; Media Cybernetics).

### Sound emission

Sound emissions were made using an underwater loud speaker (AQUA 30, DNH, 8 Ohms, 80–20,000 Hz) associated with an amplifier (Plug & Play 12 W) connected to a PC that continuously replayed vessel noise. Sound recording calibrations under experimental conditions were made using a digital recorder (Song Meter SM2+, Wildlife acoustics) connected to a calibrated hydrophone (HTI-96 MIN, High Tech, Inc.). The source was located in front of the platform. Consequently, the first row of jars was located 6 cm from the center of the source while the last was 32 cm away. The multiple reflections off the sides of the aquariums produced homogeneous sound conditions (SEM: ±1.5 dB) over the jars, which was confirmed by sound measurements performed in each jar prior the experiment. Correction function was calculated from 30 s recordings of a calibrated sound done in each jar and applied to the vessel noise to replicate as best as possible the shape of the *in situ* spectrum of the vessel noise. By varying the gain of the amplifier, the intensity was adjusted to match to natural conditions (Sound Level SL: 130 dB re 1 μPa between 100 and 10,000 Hz). Two recordings were also made in adjacent basins to check “silent” conditions (located at 15 cm and 60 cm away).

### Picoplankton assimilation experiment

*N. oculata* and *Synechococcus* sp. cultures were supplemented with 1 mM sodium [^13^C] bicarbonate (NaH^13^CO_3_, 99%) (Cambridge Isotope Laboratories, MA, USA) then transferred into three sealed 2.8 l polyethylene Erlenmeyer (Nalgen, ThermoScientific, US) and purged with nitrogen gas to eliminate atmospheric CO_2_. O_2_ generated during culture growth was removed daily and cultures were maintained at 22.5 °C with continuous orbital agitation (120 rpm) and illumination (100 μE m^−2^ s^−1^, Multitron II incubator, Infors-HT, Switzerland). To test ^13^C enrichment, 15 ml of microalgae culture were stored at −80 °C. These enriched microalgae cultures were introduced in 2.8 l Erlenmeyer, three with a unique diet of *N. oculata* (75 cells μl^−1^) and three with *Synechococcus* sp. (150 cells μl^−1^) distributed at similar biomass. In each Erlenmeyer, 20,000 pediveliger mussel larvae were added. The six Erlenmeyer were placed under natural photoperiod for 67 h. In parallel, triplicate of Erlenmeyer containing 20,000 larvae were kept as control of non-labeled and not fed larvae. At the end of the experiment, the solution of each Erlenmeyer was then filtered on glass-fiber filters GF/C 47 mm and rinsed well with distilled water to eliminate food. Larvae were gently collected by scraping the filter with a scalpel and stored at −80 °C.

### Fatty acids labeling analyses

Assimilation of microalgae was determined through the analyses of isotopic labeling levels of fatty acids (FA) in the food (picoplankton culture) and in the larvae fed with these species^64^,^65^. Samples were lyophilized, weighed and lipids extracted by a modified Folch procedure^66^ as described in Parrish *et al*.^67^ and separated into neutral (including triglycerides, free FA, and sterols) and polar (mainly phospholipids) fractions^68^. Polar fractions were hydrolyzed and the extracted FA were analyzed with a modified (see supplementary material) liquid chromatography mass spectrometry method^69^ using Fourier transform detection (LC-FT-MS, Orbitrap, XL Discovery, Thermo Scientific) in negative modes. The calculation of the percentage of isotope label for a given fatty acid is based on the ratio of the peak area of each isotopomer relative to the sum of all possible isotopomer signals using similar calculation method developed by Leblanc *et al*. for amino acids^65^.

### Data analysis

Hereafter, treatments will be identified as: S^+/−^ for sound treatment differentiation as well as Nanno^+/−^ and Syneco^+/−^ for trophic treatment with *N. oculata* and *Synechococcus* sp., respectively. Data are given as mean± the standard error of the mean (SEM). The settlement data are expressed in percentage of the larvae attached to the total number of larvae in each jar. The metamorphosis data are expressed in percentage of the larvae in prodissoconch II stage to the total number of larvae in each jar. Differences in the larval density, settlement percentage, larvae size and percentage of metamorphosis among treatments were compared using one or two way ANOVA or with a Student t-test when only two mean were compared after verification of normality and equality of variance (by Shapiro-Wilk test). Post-hoc comparisons (Tukey’s pairwise multiple comparison procedure; α = 0.05) were performed to test for any differences among individual treatments. All analyses were performed using software Systat V12.02. Enrichment level of different the dominant FA of picoplankton cultures and larvae fed with these picoplankton have been analyzed by permutational multivariate analysis of variance (PERMANOVA) using PRIMER v6.1.12^70^ with PERMANOVA+v1.0.2^71^. Homogeneity was evaluated using the permutation analysis of multivariate dispersion (PERMDISP) routine and pair-wise multiple comparison tests were used to identify differences among factors.

## Additional Information

**How to cite this article**: Jolivet, A. *et al*. Validation of trophic and anthropic underwater noise as settlement trigger in blue mussels. *Sci. Rep.* **6**, 33829; doi: 10.1038/srep33829 (2016).

## Supplementary Material

Supplementary Information

## Figures and Tables

**Table 1:** Sound level (in dB re 1 μPa) measured: *in situ* with the ambient underwater noise corresponding to the average of 24 sequences of 30 s recording each hour, and the vessel noise recorded during the three passages of the boat; before the experiment in the three aquariums; and during the experiment in the Aquarium 1 corresponding to the sound treatment and in the two adjacent aquariums without sound emission.

	100–1,000 Hz	1,000–10 000 Hz
*In situ*		
Ambient underwater Noise	86 ± 3	92 ± 2
Vessel noise *in situ* (3 passages)	130 ± 2	123 ± 2
Before experiment		
Aquariums	100 ± 5	87 ± 6
During experiment		
Vessel noise emitted in Aq. 1 (10 jars)	127 ± 3	113 ± 5
Aquarium 2	110	93
Aquarium 3	111	95

**Table 2:** Larval size (μm, mean ± SEM) and metamorphosis success (Met. in %) estimated at the end of experiment for each condition: sound treatment (Silent and vessel noise) and trophic treatment with supply of *Nannochloropsis oculata* (noted Nanno^+^/Nanno^−^) or supply of *Synechococcus* sp. (noted Syneco^+^/Syneco^−^).

	Swimming		Settled		
	Size	Met.	Size	Met.	
Silent Aquarium					
Nanno −	286.1 ± 1.3	*1* ± *1*	303.1 ± 4.2	*37.3* ± *14.4*	*a*
Nanno +	285.5 ± 1.3	*0* ± *0*	297.5 ± 2.9	*43.8* ± *13.6*	*b*
Syneco −	282.0 ± 2.9	*1* ± *1*	299.7 ± 2.9	*48.3* ± *17.2*	*b*
Syneco +	287.2 ± 1.1	*0* ± *0*	289.8 ± 5.0	*40.4* ± *4.3*	*b*
Noisy Aquarium					
Nanno −	284.0 ± 1.7	*2.5* ± *2.5*	289.0 ± 3.0	*42.4* ± *7.4*	*c*
Nanno +	284.4 ± 1.9	*0* ± *0*	297.0 ± 2.2	*59.1* ± *3.5*	*b*

Different letters in the last column indicate significant differences.

**Table 3:** Percentage of ^13^C on dominant fatty acids from enriched *Nannochloropsis oculata* (*Nanno*) and *Synechococcus* sp. (*Syneco*) and on pediveliger larvae fed with such enriched microalgae during 72 h.

	16:1	18:1	18:3	20:1	20:5	22:2nmi	
^13^C *Nanno*	98.7 ± 0.3	98.5 ± 0.8	96.9 ± 0.1	98.5 ± 0.2	98.1 ± 0.5	—	a
^13^C *Syneco*	97.8 ± 0.4	96.5 ± 0.6	90.3 ± 0.3	87.1 ± 0.1	92.1 ± 0.2	—	a
Larvae fed with ^13^C *Nanno*	25.1 ± 0.3	23.6 ± 0.1	21.3 ± 0.1	23.0 ± 0.1	23.1 ± 0.1	13.5 ± 0.1	b
Larvae fed with ^13^C *Syneco*	1.3 ± 0.1	1.2 ± 0.1	1.3 ± 0.1	2.1 ± 0.1	2.1 ± 0.1	1.5 ± 0.1	c

Different letters in the last column indicate significant differences.
