# Supplementary material for: Validation of trophic and anthropic underwater noise as settlement trigger in blue mussels
Source: Sci Rep. 2016 Sep 20;6:33829. doi: 10.1038/srep33829 (PMC5028714; doi:10.1038/srep33829)
Supplement: Supplementary Information [file srep33829-s1.pdf]

Validation of trophic and anthropic underwater noise as settlement trigger in blue mussels

Aurélie Jolivet, Rejean Tremblay, Fréderic Olivier, Cédric Gervaise, Rémi Sonier, Bertrand Genard, Laurent Chauvaud

Supporting information - Methods

Protocol for fatty acids labeling analyses

The analyses of isotopic labeling levels of fatty acids in larvae feed with the ^{13}C labeled picoplankton species was performed using a liquid chromatography mass spectrometry method using Fourier transform detection (LC-FT-MS) close to protocol developed by Kamphorst et al.[60] and Le Blanc et al.[61]. Before FT-MS analyses, each microalgae and larvae samples have been centrifuged, rinsed, lyophilized and weighed. For larvae, lipids were extracted in dichloromethane–methanol using a modified Folch procedure[62] and separated into neutral (including triglycerides, free fatty acids, and sterols) and polar (including mainly phospholipids) fractions by silica gel (30 × 5 mm, packed with Kieselgel 60, 70-230 mesh; Merck, Darmstadt, Germany) hydrated with 6% water and eluted with 10 mL of dichlormethane:methanol (98:2 v/v) for neutral lipids followed by 10 mL of methanol for polar lipids[63]. Lyophilized microalgae and dried (under nitrogen flow) polar lipids from larvae were hydrolyzed in 100 µl of KOH (9M) during 30 min at 60°C under nitrogen atmosphere. After hydrolyze, 900 µl of acetonitrile: ammonium formate (22.5 mM, pH 3.0) (3:1 v/v) were added to samples, then the solution were mixed roughly and centrifuged (5000 rpm, 5 min) at room temperature. A fraction of 600 µl of supernatant (containing fatty acids) was transferred into HPLC amber vial. 25 µl of supernatant were injected for LC-FT-MS analysis. The LC-FT-MS system consisted of an Accela UPLC pump and autosampler (Thermo Scientific, San Jose, CA), coupled with a XL Discovery Orbitrap mass spectrometer (Thermo Fisher Scientific, San Jose, CA). All components of the

system were controlled via the Xcalibur 2.1 software. The separation was performed on a Luna C18 reversed-phase column (150x2.0mm, 5 µm particle size, 100 Å pore size, Phenomenex, Torrance, CA) using a binary gradient, with solvent A composed by 100% acetonitrile with 0.01% (v/v) of formic acid and solvent B constituted by 10mM acetate ammonium in water. The injection was performed at 60% A and hold for 5 min after which the gradient increased linearly from 60 to 99% A from 5 to 7 min, remained at 99% A until 13 min and decreased quickly to 60% A at 13.1 min to remain steady at 60% until 15 min, allowing re-equilibration of the column. The flow rate was 400 µL/min, the temperatures of the autosampler and the column were 20 °C and 40 °C, respectively. The mass spectrometer was operated in negative mode. The electrospray settings for gas flow rate (arbitrary units) were: sheath gas flow rate at 25, auxiliary gas flow rate at 2 and sweep gas flow rate at 2. Finally, the spray voltage was settled at 3 kV and capillary temperature at 375°C. All raw data was processed with Xcalibur 2.1 software for signal integration. The area measured for each isotopomer was divided by the sum of areas for all possible isotopomers (up to fully labeled) of a given fatty acids. The resulting ratio (Leblanc et al. 2012) was expressed as a percentage in order to obtain a measure of the relative contribution from each isotopomer to the total amount of fatty acids as shown in the following equation:

$$\% \text{ contribution of isotopomer (C)} = \left(\frac{A_{FAn}}{\sum_{n=0}^{N} A_{FAn}}\right) X\ 100$$

where A represents the area of the FAn. FAn is the isotopomer from fatty acid 'FA' with 'n' labeled atoms, and N is the maximum number of labeled atoms for fatty acid 'FA'. From this data, the total labeling percentage was calculated by weighting each isotopomer according to the number of labeled atoms they contain, then dividing by the maximum number of labeled atoms, which corresponds to the atom percent (AP) labeled, as shown below:

$$\text{labeled AP (of fatty acid)} = \frac{\sum_{n=0}^{N} (C_{FAn})n}{N}$$
